# A Socially Aware Routing Based on Local Contact Information in Delay-Tolerant Networks

**DOI:** 10.1155/2014/408676

**Published:** 2014-07-01

**Authors:** Chan-Myung Kim, Youn-Hee Han, Joo-Sang Youn, Young-Sik Jeong

**Affiliations:** ^1^Advanced Technology Research Center, Korea University of Technology and Education, Cheonan 330-708, Republic of Korea; ^2^Department of Multimedia Engineering, Dong-Eui University, Busan 614-714, Republic of Korea; ^3^Department of Multimedia Engineering, Dongguk University, Seoul 100-715, Republic of Korea

## Abstract

In delay-tolerant networks, network topology changes dynamically and there is no guarantee of continuous connectivity between any two nodes. These features make DTN routing one of important research issues, and the application of social network metrics has led to the design of recent DTN routing schemes. In this paper, we propose an efficient routing scheme by using a node's local contact history and social network metrics. Each node first chooses a proper relay node based on the closeness to the destination node. A locally computed betweenness centrality is additionally utilized to enhance the routing efficiency. Through intensive simulation, we finally demonstrate that our algorithm performs efficiently compared to the existing epidemic or friendship routing scheme.

## 1. Introduction

A delay-tolerant network (DTN) [1, 2] has promised to enable communication between challenged networks, which includes deep space networks, sensor networks, and mobile ad hoc networks. In DTNs, network topology changes dynamically, and the lack of end-to-end connectivity poses a number of challenges in routing in DTNs. To enable communication in DTNs, messages may have to be buffered for a long time by intermediate nodes, and the mobility of those nodes must be exploited to bring messages closer to their destination by exchanging messages between nodes as they meet. So the message delivery probability is unpredictable. Designing efficient routing protocols in DTNs can tackle various issues arising due to lack of continuous network connectivity.

DTN message routing schemes have been generally made by adopting various heuristics [3–9]. They have tried to balance the overhead caused by redundant message copies with successful delivery and minimal delay of message delivery. Utilizing social network characteristic [10] has been recently studied in designing efficient routing protocols. Many studies have shown that nodes tend to have mobility patterns influenced by their social relationships and/or social behavior [1, 11]. By examining the social network of the DTN nodes, it may be possible to optimize data routing by forwarding data to nodes that are much socially related.

In DTNs, nodes encounter other nodes and store contact information (e.g., when they met and when they are separated) to their buffers. Thus, many studies have examined contact information when they analyze social relationships of DTN nodes. Bulut and Szymanski [12] introduced a new metric, called social pressures metric (SPM), to detect the quality of friendships of each node accurately. They calculated the edge weight based on contact information and used it when constructing the friendship community where the set of nodes have close friendship between each other. They also presented a new sociality-based routing scheme, called* friendship routing*, which utilizes the edge weight to make the forwarding decisions of messages.

If a sender node could know which node is important to utilize as a relay node, it would forward messages to such node to increase overall routing efficiency. Node centrality analysis is about identifying the most important nodes in a network. The betweenness centrality examines the extent to which a node is between all other nodes within the network [13–15]. Because a node with high betweenness has the capacity to facilitate interactions between nodes, it has been frequently used to design efficient data forwarding and dissemination schemes in DTNs.

On the other hand, there have been some efforts [14, 17–20] to provide an approximation of the real betweenness by using a node's local topology information. The* ego network* is the network consisting of a single node together with its immediate neighbors and all the links among those nodes. The* ego betweenness* is simply the centered node's betweenness in the ego network. It can be calculated locally by each node in a distributed manner without the complete knowledge of entire network. In our previous study [16], we examined the relationship between the ego betweenness and the globally computed traditional betweenness. We generated Bernoulli networks and calculated each node's ego betweenness and traditional betweenness on them and revealed that the relative ranks ordered by the two betweenness values have high positive correlation. It means that two nodes can compare their own locally calculated ego betweenness and the relatively higher betweenness node can be determined without the calculation of high complexity.

In this paper, we propose an efficient DTN routing scheme where each node chooses a proper relay node based on its local contact history. In order to enhance the routing efficiency, the expanded ego-network betweenness centrality [16] is utilized. In this paper, we propose how each node gets the information to calculate the expanded ego-network betweenness and identifies a proper relay node within the network. We have demonstrated that our scheme performs efficiently compared to the existing epidemic or friendship routing scheme.

The rest of this paper is organized as follows.  Section 2 provides related works dealing with socially aware routing scheme.  Section 3 explains how to construct a social network in each node and how to get the expanded ego betweenness centrality, and Section 4 presents the proposed routing scheme.  Section 5 shows a simulation analysis, and Section 6 finally concludes this paper.

## 2. Related Work

Due to the lack of global knowledge of the network topology and unstable end-to-end path in DTNs, the message routing schemes are generally made by adopting various heuristics, such as forwarding a number of message copies epidemically [3], controlled forwarding (or spraying) [4], utility-based forwarding (or estimating the likelihood of forwarding messages) [5], utilizing the contact locations [6], and focusing on the contact frequencies [7]. Such schemes were adapted over time to address different performance measures, delivery ratio, message latency, and overhead. They have tried to balance the overhead caused by redundant copies with successful delivery and minimal delay. Accordingly, multiobjective optimization is needed to solve the trade-off problems.

Recently, many researches have shown that users tend to have mobility patterns influenced by their social relationships and/or by their attraction to physical places that have special meaning with respect to their social behavior [11]. The social relations achieved by the complex network analysis may capture the inherent characteristics of the network topology and are less volatile than the transmission links (or physical contacts) between nodes. Accordingly, the application of social network analysis to DTNs has led to the design of a new class of DTN routing schemes. Hui et al. [13] proposed the BUBBLE Rap scheme where each node was assumed to have two rankings, global and local. While the former denotes the centrality of the node in the entire society, the latter denotes its centrality within its own community. Messages are forwarded to nodes having higher global ranking until a node in the destination's community is found. Then, the messages are forwarded to nodes having higher local ranking within destination's community. Daly and Haahr [14] proposed the SimBetTS scheme where the betweenness centrality metric as well as the similarity metric is used to increase the performance of routing. In each contact of two nodes, the utility function containing these two metrics is calculated for each destination; then the node having higher utility value for a destination is given the messages.

Finally, Bulut and Szymanski [12] introduced a method of detecting the quality of friendship by calculating the social pressure metric (SPM) from contact graphs. By recording contacts seen in the past, a* contact graph* can be generated where each vertex denotes a DTN node and each edge represents one or more past contacts between two nodes. An edge in this contact graph indicates the information that two nodes encountered each other in the past. Thus, the existence of an edge intends to have predictive capacity for future contacts. Bulut and Szymanski calculated the edge weight based on contact graph and used it to construct the friendship community of which nodes have close friendship between each other. They also presented a new sociality-based routing scheme, called friendship routing, which utilizes the edge weight to make the forwarding decisions of messages.

## 3. Local Information-Based Social Network Construction

In this paper, we consider a network constituted by nodes with mobility, so the network topology changes dynamically. In a DTN node, the time is discretely slotted from the start time *t*_0_ until the end time *t*_*π*_ (i.e., each time slot is denoted as *t*_0_, *t*_1_, *t*_2_,…, *t*_*π*_). Each node maintains its own clock time but shares the same length of time slot. Time synchronization is not required strictly, but a sophisticated synchronization scheme may help construct more precise ego or x-ego network. A DTN node also sends* Hello* broadcast message periodically with interval of *τ* time slots. In this paper, we assume that each Hello message contains the following information: (1)* its own identifier* and (2)* a set of identifiers of social neighbor nodes*, the latter of which will be explained later.

Let us assume that *r* is a transmission range of a node. We assume that when a node sends a message to any node within a distance *d* ≤ *r* without any failure. A node *i* can start to* contact* with (or encounter) another node *j* when *i* comes close to *j* and receives a first hello message broadcasted by *j*. Assuming that each contact lasts one time slot (i.e., each contact starts and ends during the same time slot),* a contact between two nodes i and j at time t*_*o*_ is defined as a 3-tuple 〈*i*, *j*, *t*_*o*_〉. If *i* stays within the transmission range of *j*, it can hear a periodic hello message from *j*. When *i* does not hear a predefined number of *j*'s hello messages continuously, it considers that it* leaves j*. When *i* meets *j* at a time *α* and leaves *j* at a time *β*, we define *β* − *α* as *i*'s* contact duration* for *j*.

### 3.1. Social Network Construction

Each DTN node records its contact duration information per node which it has encountered. Each node allocates* contact window* in its buffer. Its size is called* contact window size* (*ω*) which is predetermined by a node. When time goes by, the time window slides by *δ* time slots (i.e., *δ* is also called as step size). Let *T*_0_, *T*_1_,… denote the ordered sequence of time windows maintained by a node and let the *x*th time window *T*_*x*_ be defined as follows:

(1) $$\begin{array}{c} T_{x}=\left\{t_{\delta \cdot x},t_{\delta \cdot x+1},t_{\delta \cdot x+2},\ldots ,t_{\delta \cdot x+\omega -1}\right\}, \end{array}$$

where 0 ≤ *x* ≤ ⌊(*π* − *ω* + 1)/*δ*⌋.

For a time window *T*_*x*_, a node *i* maintains the contact duration time *d*_*i*,*j*,*x*_ for each node *j* which the node *i* makes a contact with during *T*_*x*_. By the definition of contact, *d*_*i*,*j*,*x*_ is set to the number of contacts (i.e., the number of 3-tuples 〈*i*, *j*, *t*_*o*_〉 where *t*_*δ*·*x*_ ≤ *t*_*o*_ ≤ *t*_*δ*·*x*+*ω*−1_) accumulated in *T*_*x*_ and it is used to determine its* social network *SN_*i*,*x*_, which is valid after the last time slot *t*_*δ*·*x*+*ω*−1_ of *T*_*x*_. In SN_*i*,*x*_, each vertex corresponds to nodes which the node *i* has encountered frequently and each edge corresponds to the relation between the node *i* and the frequently encountered nodes. We use the same edge weight as the one proposed by [12]. That is, between the nodes *i* and *j*, the following weight *w*_*i*,*j*_ is allocated:

(2) $$\begin{array}{c} w_{i,j}=\frac{\omega }{\int_{t=0}^{\omega }f\left(t\right)dt}, \end{array}$$

where *f*(*t*) returns the remaining time to the first encounter of the node *j* after time *t*. For example, let us assume that the contact window size *ω* is 10. If the node *i* is in contact with the node *j* in the first 5 seconds, then separated away for 3 seconds and contacting the node *j* for 2 seconds again, the weight *w*_*i*,*j*_ of the two nodes is 10/(3 + 2 + 1) = 5/3.

Only if the weight *w*_*i*,*j*_ is larger than the predefined threshold *θ*, an edge between the nodes *i* and *j* is created between the two nodes in the node *i*'s social network SN_*i*,*x*_. Usually, *θ* is empirically determined depending on the contact window size *ω* as well as the scenario of network operation. For a time window *T*_*x*_, we will denote* a node i's social neighbor nodes* by *N*_*i*,*x*_.

It is noted that a DTN node's social network is constructed by using the information about a neighbor's neighbor nodes as well as just neighbor nodes. In order to support such a social network, a DTN node should piggyback *N*_*i*,*x*_ determined at every end of *T*_*x*_ into the Hello messages sent during *T*_*x*+1_. Since the social neighbor nodes are determined during the previous time window, its information will be more fresh if the step size *δ* of time window and the Hello message interval *τ* are small. At every end of time window *T*_*x*_, a node *i* creates its expanded ego network by its neighbor node *j*'s neighbor nodes *N*_*j*,*y*_ where *y* represents the index of the time window *T*_*y*_ maintained by the node *j*.

Algorithm 1 represents the social network construction procedure for a node *i* where *V*_*i*,*x*_ and *E*_*i*,*x*_ mean the sets of nodes and edges in SN_*i*,*x*_. Note that the algorithm is performed every end of contact window.

### 3.2. Expanded Ego Betweenness Centrality

The betweenness centrality has been used as important measure to examine the extent to which a node is between all other nodes within the network [15]. When the message is forwarded to nodes with high betweenness centrality, that message can be disseminated to entire network in fast way. In this paper, we will use the betweenness centrality to increase overall routing efficiency. In a DTN, however, obtaining the betweenness of each node is in general impractically expensive since it requires DTN nodes, which usually have limited memory and energy, to collect information about their whole social links through wireless communications. In this paper, therefore, we consider the situations where each node computes its betweenness using its local expanded ego network and then uses the result as an estimate of its true betweenness on the entire network.

For an arbitrary node *i*, the equation of betweenness centrality *C*_*B*_(*i*) is defined as follows:

(3) $$\begin{array}{c} C_{B}\left(i\right)=\underset{s\neq i\neq t\in V,s<t}{\sum }\frac{\rho_{st}\left(i\right)}{\rho_{st}}, \end{array}$$

where *V* is the set of nodes in the network and *n* is the total number of nodes, *ρ*_*st*_ the number of shortest paths between the two nodes *s* and *t*, and *ρ*_*st*_(*i*) is the number of those shortest paths that pass through the node *i*. As mentioned previously, the betweenness centrality requires the entire network information but a node cannot know it due to lack of the whole network-wide end-to-end connectivity.

As we saw in previous section, a node constructs its social network with its friends and friends of friends information. Hence, we use the expanded ego betweenness proposed by [16], which is calculated only with its local contact information.

**Figure 1:**
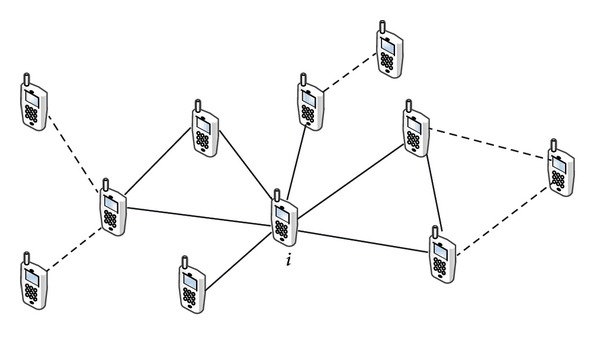
Node *i*'s expanded ego network [16].

Figure 1 illustrates an expanded ego network of a node *i*. The network is constituted by the ego (the node *i*), its 1-hop neighbors, and its 2-hop neighbors. While the solid links present the* ego network* introduced in literature [18, 21], the solid and dashed links represent the* expanded ego network* which is constructed by Algorithm 1. In short, the* expanded ego betweenness centrality* of the node *i* is equal to the betweenness centrality of the node *i* within its expanded ego network. In our previous work, we verified that the expanded ego betweenness centrality is highly correlated with the betweenness centrality in the complete network. For the details of the expanded ego betweenness centrality, refer to [16].

## 4. Routing Strategy

In our algorithm, a node (1) calculates the weight based on the contact history recorded for other nodes it has encountered, (2) constructs its own social network, and (3) calculates the expanded ego betweenness centrality. With these pieces of information, a node makes a decision on a relay node when it tries to send a message to the destination node.

### 4.1. Edge Weight Based Strategy

**Figure 2:**
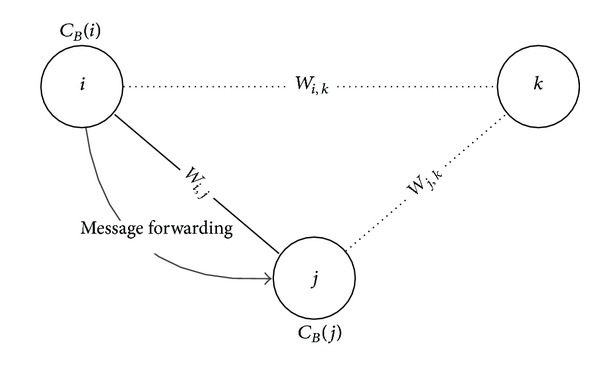
Message forwarding condition: *w*_*j*,*k*_ > *w*_*i*,*k*_ or *C*_*B*_(*j*) > *C*_*B*_(*i*).

Figure 2 depicts a situation where a node *i* tries to send a message to the remote destination node *k* and it just contacts a node *j*. The node *i* tries to make a decision that it should forward (i.e., copy) the message to the node *j*. Basically, at this time, the node *i* considers the two edge weight values *w*_*i*,*k*_ and *w*_*j*,*k*_. A high edge weight between a pair of two nodes represents how close they are and it indicates that the future contact opportunity comes high. In our scheme, the node *i* forwards the message to the node *j* if the following condition is met:

(4) $$\begin{array}{c} \text{Condition}\;\;\text{I:}\;\;w_{j,k}>w_{i,k}. \end{array}$$

This strategy is similar to the one proposed by [12].

### 4.2. Centrality Based Strategy

On the other hand, we use the expanded ego betweenness centrality to increase message delivery efficiency. In DTNs, some nodes hardly meet other nodes and they have very low edge weights for the previous encounters. If one of these isolated nodes is set to be the destination node, source node might not find a proper relay node to deliver a message, when it would forward the message only by using the edge weight based strategy. In such situation, the message destined to the isolated node could be removed from the sender's buffer by TTL (time-to-live) expiration before delivered to the destination.

To prevent this situation, a node *i* also forwards a message to a node *j* if *C*_*B*_(*j*) is larger than *C*_*B*_(*i*) even though Condition 1 is not met. That is, the second condition for the message forwarding is as follows:

(5) $$\begin{array}{c} \text{Condition}\;\;\text{II:}\;\;C_{B}\left(j\right)>C_{B}\left(i\right). \end{array}$$

A high expanded ego betweenness centrality of a node represents that the node is much socially related with many other nodes. Hence, the message forwarding to such a node makes the opportunity high that the message will reach the destination node, and thus such forwarding strategy can increase the overall efficiency of message delivery.

### 4.3. Message Delivery Cost Reduction

In this section, we propose a message management scheme in a node's buffer to decrease the overall delivery cost. If a node *i* has a message in its buffer destined to the node *k* and encounters a node *j* which satisfies *w*_*j*,*k*_ > *w*_*i*,*k*_ (Condition I), the node *i* will forward the message to the node *j*. In this case, if the edge weight *w*_*j*,*k*_ is the largest among the edge weights between other nodes in SN_*i*_ and the node *k*, the node *i* deletes the message from its buffer after forwarding the message to the node *j* to prevent further dissemination. That is, the node *j*, the node *k*'s best friend, will take on the role of the message delivery to the node *k*, instead of the node *i*. This strategy will reduce the number of message deliveries and enhance the efficiency of the proposed scheme.  Algorithm 2 represents our overall message routing strategy.

## 5. Performance Evaluation

In this chapter, we demonstrate our simulation results and compare the proposed scheme with the epidemic [3] and the friendship [12] routing schemes. We use the following three metrics to evaluate our scheme: (1) message delivery ratio, (2) message delivery cost, and (3) message delivery efficiency. The delivery ratio is the proportion of messages delivered to their destinations among the total messages generated. The delivery cost is the average number of forwards done during the simulation. Finally, delivery efficiency is defined as the ratio of delivery ratio to the delivery cost.

To evaluate our scheme, we used real trace-driven simulations based on predefined node mobility data. From the mobility data, we generated the contact trace data logged during the simulation time. The trace data were converted into discrete sequential contact events per unit time per each node pair, and we fed them into each DTN node implemented in our evaluation program. In the evaluation program, each node created a series of sets of contacts where each set corresponds to each contact window. Every end of contact window, each node computed the cumulative contact duration for each of contact nodes and determined its social neighbors of which cumulative contact duration larger than the given threshold (i.e., *θ*), constructed its expanded ego network and finally computed its expanded ego betweenness on the expanded ego network.

As the first contact window time passed, all nodes started to generate 1000 messages totally. Each message was destined from a random source node to a random destination node. Each message had a certain TTL value and was removed after the TTL expiration. The simulation ended when the 1000 messages are delivered to the destination or expired. All results were averaged over 10 runs.  Table 1 summarizes the simulation parameters.

**Figure 3:**
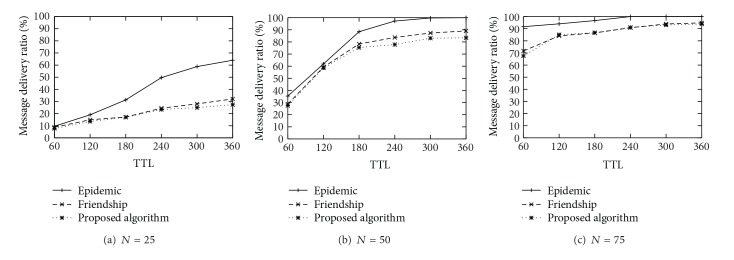
Delivery Ratio (%).

**Figure 4:**
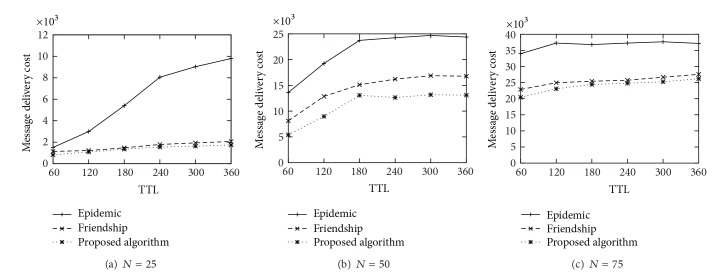
Delivery Cost.

Figure 3 shows the delivery ratio achieved by each schemes. As the TTL increases, all schemes deliver more messages to the destinations. As expected, the epidemic routing scheme has the highest packet delivery ratio. It is noted that the delivery ration of proposed scheme is almost similar to the one of friendship routing scheme.  Figure 4 shows the message delivery cost of each scheme. We can observe that the epidemic scheme has the worst performance in terms of the delivery cost because it uses the flooding strategy basically. It is worth noting that the proposed scheme is more cost effective than the friendship scheme, not to mention the epidemic scheme. It is because our routing scheme uses the centrality based routing strategy as well as the edge weight based strategy, and it also removes the message from a node's buffer to prevent imprudent dissemination.

**Figure 5:**
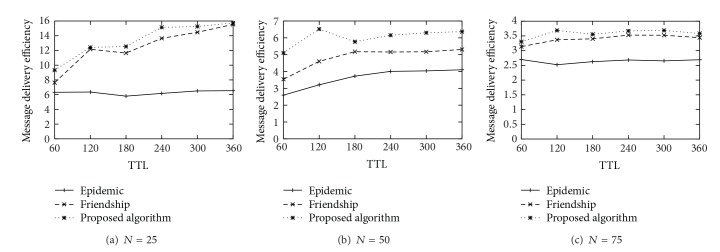
Delivery efficiency.

Finally, Figure 5 shows the message delivery efficiency achieved by each scheme. As can be seen by the figure, the routing efficiency achieved by the proposed scheme is higher than the one of other schemes. It means that our scheme has the benefit of cost effective routing with a little performance degradation of delivery ratio.

## 6. Conclusion

In this paper, we introduced a cost effective routing scheme that uses its local contact history. Each node constructs its social network based on the local contact history and calculates the expanded ego betweenness centrality on the social network. Each node performs the local routing scheme based on two metrics, edge weight and betweenness centrality. It also tries to reduce message delivery cost by clearing out the message from its buffer after it delivered it to the destination node's best friend. We simulated our scheme and compared its performance with the existing epidemic and friendship scheme's ones. We have shown that our scheme achieves higher delivery efficiency than the existing schemes. Since most of nodes in DTNs are energy constrained, we plan to further examine energy-efficient routing based on social network in DTNs.

## Figures and Tables

**Algorithm 1:**
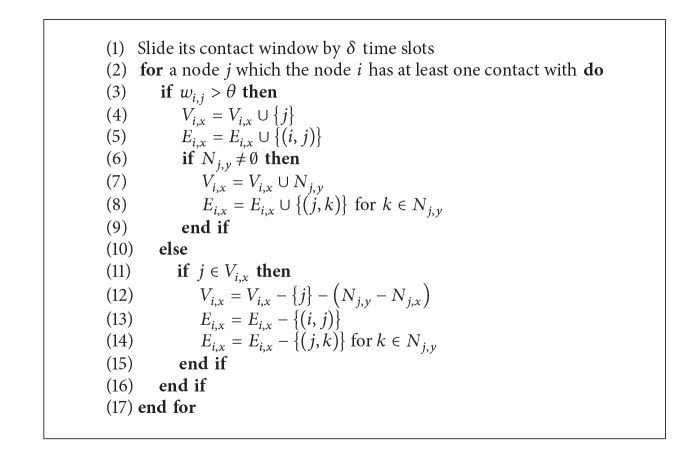
Social network construction procedure for a node *i*.

**Algorithm 2:**
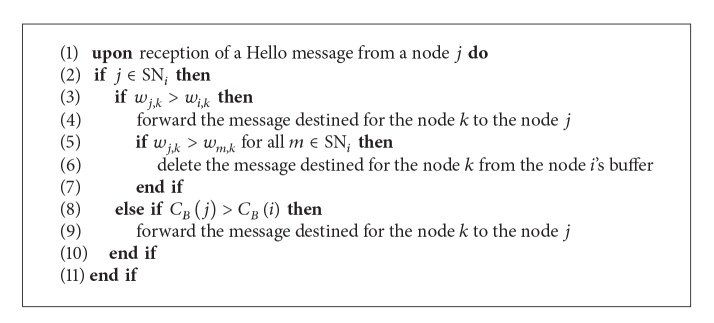
Message forwarding procedure when a node *i* tries to deliver the message to the destination node *k*.

**Table 1:** Simulation parameters.

Area size	1000 m × 1000 m
Number of nodes (*N*)	25, 75
Communication range	3 m
Moving speed	0.5 m/s, 1.0 m/s, 1.25 m/s, 1.5 m/s
Contact window size (ω)	600 seconds
Window sliding unit (δ)	60 seconds
Hello message interval (τ)	1 second
Threshold (θ)	0.01
Number of messages	1000
